# Airways cephalometric norms from a sample of Caucasian Children

**DOI:** 10.4317/jced.58105

**Published:** 2021-09-01

**Authors:** Luis-Miguel Pérez-Rodríguez, Montserrat Diéguez-Pérez, Alejandrina Millón-Cruz, Ignasi Arcos-Palomino

**Affiliations:** 1 DDS, MSc, PhD. School of Dentistry. Universidad Europea de Madrid. Campus de Universidad Europea de Madrid. Calle Tajo, s/n, 28670 Villaviciosa de Odón, Madrid; 2 MD, PhD. Department of Oral and Maxillofacial Surgery. Hospital Universitario Príncipe de Asturias. Carretera Alcalá-Meco s/n 28805, Madrid.

## Abstract

**Background:**

The diagnosis of the respiratory pattern and the analysis of airway dimension using lateral cephalometric radiographs include the study of the adenoid region, free air space of the nasopharynx and oropharynx, soft palate and posterior part of the tongue. The objective of this study is to identify the airways cephalometric norms from a sample of Caucasian children, in relation to gender, age and type of malocclusion.

**Material and Methods:**

A total of 480 patients of both sexes were included in the study, the age ranged between 6-12 years. The radiographic records were analyzed using the Nemoceph® 11.3.0 software and the diagnosis of skeletal class was performed using the Steiner analysis. The cephalometric measurements used for the study were PNS-AD1, AD1-Ba, PNS-Ba, Ptm-Ba, PNS-H and the upper and lower airways according to McNamara analysis. The comparative analysis was performed using only upper and lower airways variables.

**Results:**

The mean values for each variable in the total sample were 23.2 mm (PNS-Ad1), 24.7 mm (Ad1-Ba), 47.6 mm (PNS-Ba), 45.7 mm (Ptm-Ba), 30.0 mm (PNS-H), 9.3 mm (upper airway) and 11.5 mm (lower airway). According to gender, all variables were greater in the boys group except for the lower airway. In relation to age, the mean values increased with age except for the lower airway and the AD1-Ba variables. In patients with skeletal Class I greater dimensions of the upper and lower airways were observed.

**Conclusions:**

In this Caucasian sample, it has been observed a tendency of minor airway dimensions in patients with skeletal Class II, lower age range female gender. It has been observed only significant differences between age and skeletal class for lower airways variable and, in relation to upper airways variable the results were significant in relation to age.

** Key words:**Child development, Diagnostic XRay, Cephalometry, Respiratory system diagnostic imaging.

## Introduction

Nasal breathing optimizes the development of an ideal occlusion and stabilizes transverse and vertical bone alterations after orthopedic and orthodontics treatment. However, a smaller diameter of the upper airways may encourage the presence of oral breathing and influences the pattern of facial development and malocclusion (1). When the nasal airflow is restricted, the subject then diverts the breath to the oral cavity, creating a pathological condition known as mouth breathing. The airway analysis is an important tool for the morphological diagnosis, not only for the respiratory tract but also for the adenoid tissue. It provides information of skeletal anatomy, position of the hyoid and soft palate, and also provides data on the degree of respiratory obstruction (2,3).

Several authors have studied the reproducibility of airway dimensions on the lateral cephalometric radiograph, demonstrating that when the x-ray is taken in natural head position, the dimensions obtained are highly reproducible (4). Actually, there is no consensus among the different authors on what is the ideal cephalometric parameter for the airways study (5). There is currently no consensus regarding the type of cephalometric analysis used for, and they suggest different values of normality depending on the population studied.

Three research lines have shown the use of cephalometric studies for the adenoid size analysis, the influence of the respiratory pattern on facial morphology and sleep apnea. Authors such as Wildman, Engman, Bushy, Schweiger and Chieric performed cephalometric studies to assess soft tissue anatomy and its relation to skeletal landmarks (6). Solow *et al*., in the cephalometric evaluation of the airways, described and identified a series of points and lines, some of which are still current (7). Ricketts described a set of measures related to nasopharyngeal depth to determine its degree of permeability (8). According to McNamara, “adenoid facies” are not always present in mouth breathing patients and different varieties of facial types are observed (9). He introduced two measurements, which are currently relevant, in order to evaluate the upper and lower pharyngeal diameter (10).

In recent years, there has been an increase in studies of upper airways, due to the relationship between the size of these and craniofacial morphology (11,12).

The aim of this study is to identify the airways cephalometric norms from a sample of Caucasian children, using relevant airways measurements, in relation to gender, age and type of malocclusion.

## Material and Methods

A cross-sectional and randomized study was performed using radiographic records of 554 pediatric patients. The patients went to the Department of Stomatology IV, School of Dentistry in the Universidad Complutense of Madrid for orthodontics treatment. General medical data were obtained and each legal representative signed a consent form authorizing the use of the records for investigating purposes. The procedures followed were in accordance with the Helsinki Declaration of 1975, as revised in 2000.

The inclusion criteria were Caucasian children aged between 6 and 12 years, with lateral cephalometric radiograph with sufficient quality to be evaluated, skeletal Class I and II, absence of any orofacial pathology, dysmorphology, syndrome or other alterations that could cause changes in the normal development and or growth, no previous history of having received interceptive or corrective treatment of malocclusion, and who are not carriers of orthodontic appliances.

**Figure 1:**
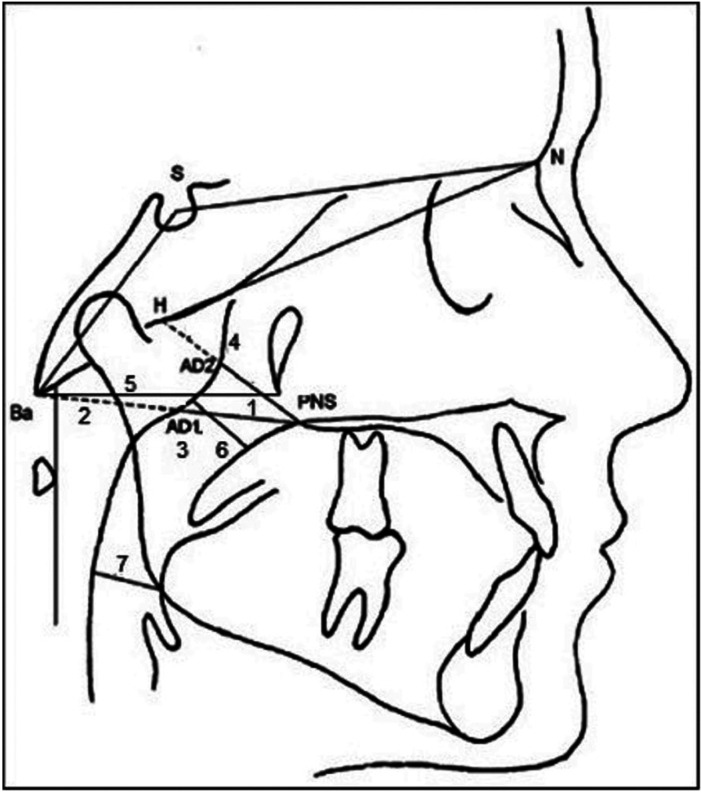
Cephalometric measurements of the airways.

All radiographic records were made with a Siemens Ortofox® model. The instructions given at the time of radiographic intake were verbal and common to all patients. X-rays were examined by the principal investigator. When duplicate records were available, the highest quality was selected. The cephalometric analysis was performed using the Nemoceph® software version 11.3.0. The records were calibrated and we proceeded to localize 47 reference points. Then, the cephalometric tracing was performed. The diagnosis of malocclusion was based on Steiner’s analysis. Angular and linear measurements were recorded in degrees and millimeters, respectively. The airways cephalometric study was based on the analysis described by Mc Namara (10) and five measures described by Mucedero (13). The studied variables were: (Fig. 1).

1. PNS-Ad1. Thickness of the lower portion of nasopharynx: distance between the posterior nasal spine (PNS) and the most proximal part of the adenoid tissue through the line formed between the posterior nasal spine and basion (Ad1).

2. Ad1-Ba. Lower adenoid thickness: distance between the posterior wall of the pharynx and basion, following the extension of the Ad1 line.

3. PNS-Ba. Lower sagittal width of the nasopharynx. It is determined by the sum of the distances PNS-Ad1 and Ad-Ba.

4. PNS-H. Total thickness of the upper part of nasopharynx. It is determined by the distance between PNS and hormion (H, hormion point, located at the intersection between the perpendicular line to S-Ba from PNS and the cranial base)

5. Ptm-Ba. Posterior sagittal width of bony nasopharynx: determined by the distance between points Ptm and Ba.

6. Upper pharyngeal diameter (upper airway). Linear measurement from the posterior wall of the soft palate to the closest point on the posterior wall of nasopharynx.

7. Lower pharyngeal diameter (lower airway): A linear measure from the intersection of the posterior border of the tongue with the lower border of the mandible to the nearest point on the posterior pharyngeal wall.

-Statistical analysis

The statistical analysis was carried out by the Statistics Department of the Complutense University of Madrid, using the SPSS 22.0 program for Windows®. Descriptive analyses of patient demographics and all the variables were summarized using means and standard deviations.

A total of 120 randomly selected cephalometric tracings were repeated to verify intra-examiner reliability and the Wilcoxon test of the signed ranges was applied, the significance level was set at *P*<0.05. No significant differences were found between the two records (*P*>0.05).

ANOVA test was used for the comparative analysis of lower and upper airways variables in relation to skeletal class, gender and age. A *P* value of < 0.05 was deemed to be statistically significant. And a decision tree method was performed for classify and segment the sample.

## Results

After applying the selection criteria, a total of 480 patients were evaluated. The sample was divided into four groups, according to sex and type of occlusion. In relation to age, the subjects were distributed in four groups of 6, 8, 10 and 12 years respectively, as shown in Table 1.

**Table 1:**
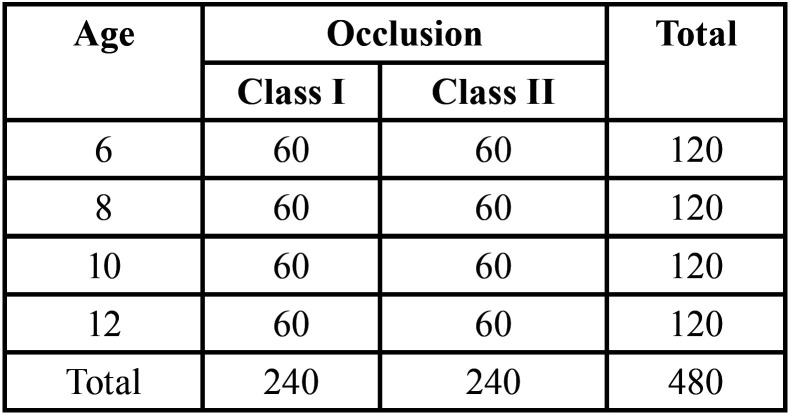
Distribution of the sample by gender, age and type of occlusion.

According to the frequency and distribution of airway permeability in the total sample, the mean values and standard deviation of each variable are shown in Table 2.

**Table 2:**
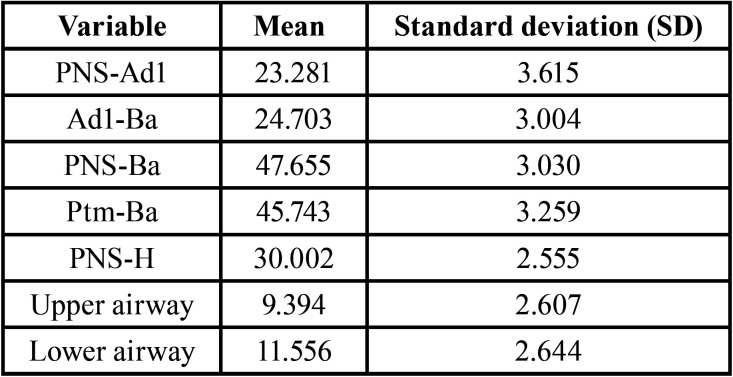
Study variables in the total sample. Mean and Standard Deviation (SD).

In relation to gender, the value obtained of the upper airway dimension was lower in the girls group (9,315 +/- 2.6 mm) compared to boys group (9,472 +/- 2.6 mm). However, the mean value of the lower airway variable was higher in the girls group (11,714 +/- 2.6 mm compared to 11,399 +/- 2.6 mm in the boys group). The value obtained for the rest variables were higher in the boys group (Table 3).

**Table 3:**
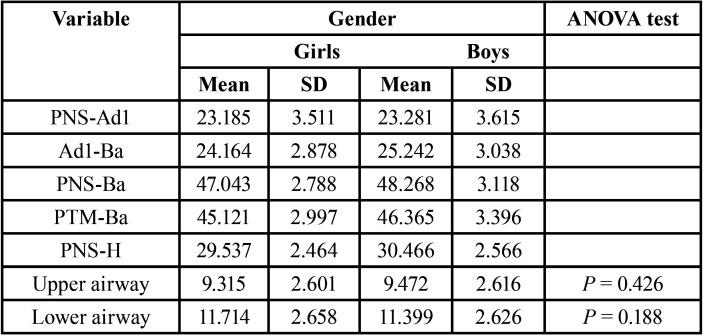
Study variables by gender. Mean and Standard Deviation (SD). ANOVA test.

Comparing the results from upper and lower airways dimensions in relation to gender, the differences observed were not significant (Table 3).

When studying the airway variables by age range, the mean value obtained for the upper airway variable was increasing in relation to age; at age 6 (7.5 mm +/- 1.82), age 8 (8.52 mm +/- 2.14), age 10 (10.11 mm +/- 2.31), age 12 (11.41 mm +/- 2.26), these differences were statistically significant (*P*< 0,05). This correlation was not observed for the lower airways variable, at age 6 (12.01 +/- 2.85), age 8 (11.42 +/- 2.66), age 10 (11.55 +/- 2.85), age 12 (11.22 +/- 2.45) and these results were not significant (Table 4).

**Table 4:**
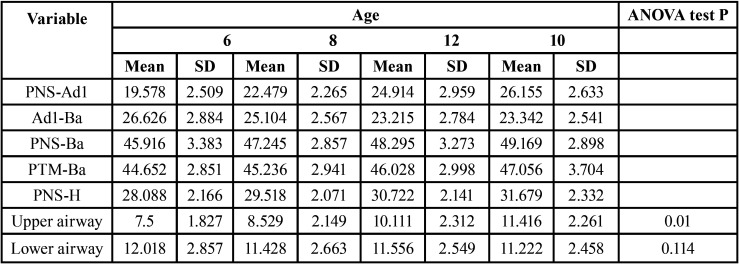
Study variables by age range. Mean and Standard Deviation (SD). ANOVA test.

Regarding the measures obtained of PNS-Ad1, PNS-Ba, PTM-Ba, PNS-H variables, these variables increased with age with except for the Ad1-Ba variable (Table 4).

Analyzing the frequency and distribution of airway permeability by skeletal class, the means in patients with Class I for the upper and lower airway variables were 9.45 +/- 2.67 and 11.66 +/- 2.55 mm respectively, these values were higher than those obtained in patients with Class II, 9.34 mm +/- 2.55 and 11.45 mm +/- 2.74 mm, respectively. In relation to Ad1-Ba and Ptm-Ba variables, the means obtained were higher in patients with Class I. But, the variables PNS-Ad1, PNS-Ba and PNS-H were greater in patients with Class II, compared to patients with Class I (Table 5).

**Table 5:**
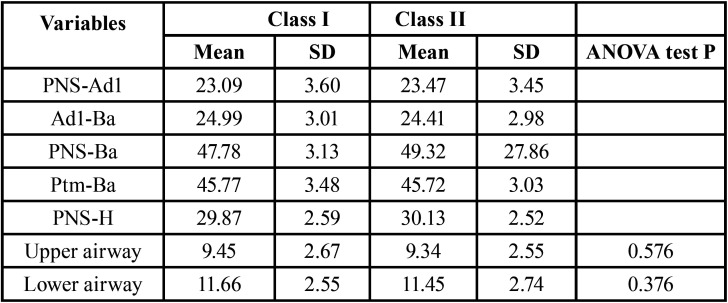
Study variables and significance by skeletal class. Mean and Standard Deviation (DS). ANOVA test.

Comparing the results obtained for the airway permeability in relation to skeletal class I and II, no statistically significant differences were observed (Table 5).

**Figure 2:**
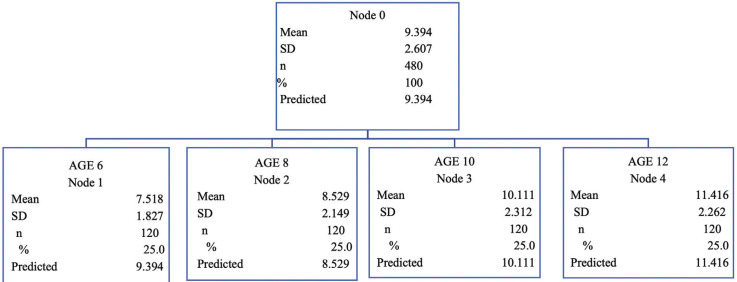
Classification tree in relation to upper airways variable and age.

In the analysis of the three variables (gender, age and skeletal class) with ANOVA test in relation to upper airways, no statistically differences were observed. But, for lower airways we only observed a statistically significant relation between age and skeletal class (Table 6). Finally, in the classification tree analysis, it has been compared age and upper airways dimension because it was the only variables which were statistically significant. A decision tree to classify children in relation to age and upper airway dimension was obtained (Fig. 2).

**Table 6:**
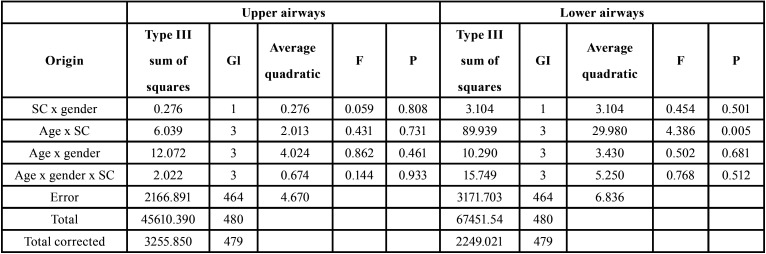
Upper and lower airways in relation to gender, age and skeletal class (SC). ANOVA test.

## Discussion

The main factors in the airways assessment are: the sagittal relationship between the maxilla and jaw, the growth pattern, related problems such as sleep apnea and hypertrophy of adenoids and / or tonsils. To determine the skeletal class, Steiner’s analysis has been used by a large number of authors to determine the type of sagittal bone relation (14).

Considering age and gender, in the study of Nuernberg and Vilella about oropharynx dimension, the normal values obtained in patients between 6 and 11 years of age were from 8.76 to 14.86 mm (15). In this research the mean value obtained in patients between 6 and 12 years old was 11.55 +/- 2.64 mm; a similar result to the Nuernberg study (15). A relation between the age and dimension of the oropharynx was not observed, but it was observed for the upper airway. In contrast, Mislik *et al*. evaluated the airway size and its physiological changes, using the analysis of lateral radiography. Their results showed a great variability in the sample; with the exception of 9-year-old children group, no statistically significant differences were found by gender. The influence of age was observed in regard to upper airway whereas in lower airway it was not relevant, concurring with this study (16). Hanggi *et al*. compared the upper airway size using lateral radiography in a sample of growing children and adolescents. The differences in growth and individual response to malocclusion treatment were observed in the study (17). In this study was observed differences in the relationship between the upper airway dimension and the age, coinciding with the conclusions of Hanggi´s study, in relation to differences which exist in growing patients. At the same time, Tsai performed a cross-sectional study to evaluate changes in the development of the pharyngeal airway from young to adult subjects. The results indicated that the depth of the upper pharynx increased with age. However, the lower pharyngeal dimension stabilized earlier. Changes in development of pharyngeal structures were significantly higher in males than in females (18). In this study the results obtained were concordant with Tsai, the size of the upper airways was increasing with age, while the inferior airway stabilized earlier. According to gender, a larger size of the pharyngeal structures was found in the boys group compared to the girls group, but it was not statistically significant. Li *et al*. carried out a 3D study by computed tomography of airways. They did not find differences between sexes at infantile age, but they found them in adolescents. They observed a significant increase of the airways size in relation to age (19). In both previous studies and in our study, most of the authors agree on the relationship between the size of the airways, age and gender.

Regarding the skeletal class, in this study, patients with Class I presented a larger airway size compared to Class II patients, both at the nasopharynx and oropharynx. These differences were non-significant, but a tendency of smaller airway size in Class II patients was observed. These results were similar to the study by Mendoza *et al*., they evaluated patients with airway obstruction and skeletal class. After the analysis of 100 radiographs, they compared the results obtained with the norms established by McNamara. A higher frequency of airway obstruction in patients with skeletal Class II was observed, and being more frequent in male (20). But the age range used by these authors was broader than in this study. Bollhalder *et al*. analyzed the relation between the upper airways and the degree of severity of Class II, observing in the retrognathic patients a tendency of smaller airways. These results agree with those obtained in this study (21). Also, this study obtained similar results to those obtained in other studies, such as Indriksone *et al*. (22).

Hakan *et al*. concluded that there was a relation between Class II patients with mandibular retrognatia and decreased airway size, so subjects with Class III and mandibular protrusion showed a larger airway dimension (23); Kirjavainen *et al*. observed that patients diagnosed of Class II division 1 presented a narrower upper airway, even without retrognathia (24). At the same time, Zhong *et al*. found a significant relation between the size of the upper airway and the sagittal and vertical relation which presented each patient (25). Similar results were found in this study regarding the sagittal relationship, although they were not statistically significant. In contrast, Freitas *et al*. observed no influence between the size of the upper and lower airways and the type of malocclusion (Class I and Class II skeletal) or the growth pattern (26). The same results were obtained regarding the skeletal class as these authors, but a tendency of smaller airway dimension was observed in Class II patients. It was observed a significant relation between age and skeletal Class in ANOVA- test. The growth pattern was not evaluated.

Alves *et al*. carried out a similar study in order to evaluate the air space in relation to the skeletal pattern using the CBCT. After establishing two groups, children who presented Class I and Class II maloclussion, they observed a significant greater amplitude of the airways in the patients with Class I (27). In this study regarding the relationship between skeletal class and airway size, a larger airway size was observed in Class I patients compared to Class II patients. These results were not statistically significant. However, a relation was found between Class I and a larger airway dimension. Kim *et al*. analyzed the dimension of the superior pharyngeal airway in healthy children and in children with retrognathic jaw, using the 3D technique. The volume of upper airway, extending from the anterior nasal cavity and the nasopharynx to the epiglottis, in retrognathic patients was significantly smaller than the patients with a normal skeletal relationship (28).

## Conclusions

1.The mean values obtained for the airways variables were: PNS-Ad1 (23.28 mm +/- 3.60), Ad1-Ba (24.70 mm +/- 3.00), PNS-Ba (47.65 mm +/- 3.03), PTM-Ba (45.74 mm +/- 3.25), PNS-H (30.00 mm +/- 2.55), upper airways (9.39 mm +/- 2.60) and lower airways (11.55 mm +/- 2.64 ).

2. Regarding the McNamara analysis, a lower value for the upper airway was obtained.

3. We observed a lower value of all the variables in girls group, except for lower airways variable. However, we did not find significant differences for upper and lower airways variables in relation to gender.

4. No significant differences were observed between the Class I and Class II groups for the upper and lower variables, also these variables were greater in Class I group.

5. Significant differences were observed in relation to age and upper airways variable, establishing a decision tree for the means in each age group.

6. In the analysis of ANOVA, it was observed that there is a significant relationship between age and skeletal class, therefore, it would be necessary to carry out a further exhautive study between the age and skeletal class groups.
